# Retinal ectopias and mechanically weakened basement membrane in a mouse model of muscle-eye-brain (MEB) disease congenital muscular dystrophy

**Published:** 2010-07-28

**Authors:** Huaiyu Hu, Joseph Candiello, Peng Zhang, Sherry L. Ball, David A. Cameron, Willi Halfter

**Affiliations:** 1 Department of Neuroscience and Physiology, SUNY Upstate Medical University, Syracuse, NY; 2 Louis Stokes Cleveland Department of Veterans Affairs Medical Center, Cleveland, OH; 3 Department of Neurobiology, University of Pittsburgh, Pittsburgh, PA

## Abstract

**Purpose:**

Some forms of congenital muscular dystrophy are associated with cortical and retinal dysplasias. Protein O-mannose N-acetylglucosaminyltransferase 1 (POMGnT1) knockout mice, one of the mouse models of muscular dystrophy, exhibit a thinner retina with reduced density of retinal ganglion cells. This study is aimed to further characterize the knockout retina, with special emphasis on the inner limiting membrane, the basement membrane of the retina.

**Methods:**

Immunofluorescence staining and transmission electron microscopy were used to analyze the retinas. Atomic force microscopy was performed on the inner limiting membrane preparations to examine their mechanical properties.

**Results:**

The inner limiting membrane of the knockout mice exhibited frequent breaks with protrusions of the Müller glial processes and ectopic placement of retinal ganglion cells into the vitreous humor. Disruptions in inner limiting membrane integrity developmentally precede the cellular abnormalities. Regions of disrupted inner limiting membrane were also associated with molecular abnormalities of Müller glia that included diminished presence of the integral membrane proteins Kir4.1 (an inwardly rectifying potassium channel) and aquaporin-4. When measured with atomic force microscopy, the POMGnT1 knockout mouse inner limiting membrane (ILM) exhibited significantly reduced Young’s modulus and is therefore mechanically weaker than the ILM from controls.

**Conclusions:**

Deficiency of POMGnT1-mediated glycosylation of dystroglycan is implicated in reduced stiffness of the ILM. The weakened ILM results in the disruption of the membrane and subsequent reduction in retinal integrity.

## Introduction

Congenital muscular dystrophies (CMDs) with type II lissencephaly and retinal malformations include Walker–Warburg syndrome (WWS), muscle-eye-brain disease (MEB), Fukuyama congenital muscular dystrophy (FCMD), and congenital muscular dystrophy 1D (MDC1D) [1-13]. Many of these patients have mutations in genes encoding glycosyltransferases (or putative glycosyltransferases), *POMT1* (encoding protein O-mannosyltransferase 1, POMT1) [14,15], *POMT2* [16], *POMGnT1* (encoding protein O-mannose N-acetylglucosaminyltransferase 1, POMGnT1) [17], *Large* [18], *FCMD* (encoding fukutin) [19,20] *FKRP,* (encoding fukutin-related protein, FKRP) [21-23]. Ocular abnormalities of muscle-eye-brain disease include a predisposition to glaucoma, progressive myopia, juvenile cataracts, nystagmus, uncontrollable eye movement, and retinal atrophy with reduced retinal function [1,9,11,24].

The mouse model of muscle-eye-brain (MEB) disease exhibits similar phenotypes in the retina. POMGnT1 knockout mice have a thin retina with reduced density of retinal ganglion cells [25]. Functionally, the knockout retina has reduced electroretinogram response in dark-adapted conditions [25]. Similar phenotypes exist in other mouse models of CMDs, the natural mutant Large^myd^ mice [26,27], and chimeric fukutin knockout mice [28].

A common molecular phenotype in these CMDs is the hypoglycosylation of α-dystroglycan, a glycoprotein heavily substituted by O-linked glycans, particularly O-linked mannosyl type, for example, Siaα2,3Galβ1,4GlcNAcβ1,2Man-Ser/Thr [29-31]. At least some of the identified CMD genes are involved in the synthesis of O-mannosyl glycans. POMT1 and POMT2 are an enzyme complex that transfers mannose to serine or threonine residues [32,33]. POMGnT1 transfers N-acetylglucosamine to O-linked mannose [17,34]. The catalytic functions of fukutin and Large are not yet fully identified. Large is involved in phosphoryl glycosylation of O-mannose and complex N- or mucin O-linked N-acetylgalactosaminyl glycans [35-37]. At the cell surface, α-dystroglycan binds with high affinity to several extracellular matrix components, including laminin, agrin, perlecan, neurexin, and pikachurin, in a manner dependent on its carbohydrate conjugates [38-43]. α-Dystroglycan binds to the transmembrane β-dystroglycan at the cell surface [44,45]. The intracellular domain of β-dystroglycan interacts with cytoskeletal components, such as dystrophin and utrophin. Thus, α-dystroglycan and its glycoconjugates participate in an important linkage between the extracellular matrix and the cytoskeleton. Hypoglycosylation of α-dystroglycan leads to loss of its binding activity to laminin, a major component of the extracellular matrix basement membrane [18,25,28,46-49], and thus would negatively affect the mechanical linkage between the basement membrane and intracellular cytoskeleton.

The basement membrane is a specialized extracellular matrix that is mainly composed of laminins, collagen IV, perlecan, and nidogen [50,51]. Laminins and collagen organize this matrix via polymerization and bind to nonpolymerizing molecules, such as perlecan. The retina has two specific basement membranes, the inner limiting membrane of the neural retina and Bruch’s membrane of the pigmented epithelium. In this paper we describe biologic and physical effects of POMGnT1-deficiency on the inner limiting membrane, with concomitant effects upon some retinal cell types.

## Methods

### Animals

Protocols for animal usage were approved by the Institutional Animal Care and Use Committee of the State University of New York Upstate Medical University and were in accordance with National Institutes of Health guidelines.

POMGnT1 knockout mice were generated from a *POMGnT1*-trapped embryonic stem (ES) cell line in the OmniBank embryonic stem cell gene-trap library in collaboration with Lexicon Genetics Inc. (The Woodlands, TX) [25]. For timed pregnancy, noon on the date of plug observation was considered embryonic day (E)0.5. Date of birth was considered postnatal day (P)0. A total of eight homozygous (−/−) fetal mice (two at E11.5, one at E13.5, three at E15.5, and two at 17.5), 11 homozygous postnatal mice (one at P0, one at P21, and nine adults) were used in this study. Wild-type (+/+) and sometimes heterozygous (+/−) littermates were used as controls.

### Antibodies

IIH6C4 (an antibody against carbohydrate epitope of α-dystroglycan), anti-water-conducting protein aquaporin-4 (AQP-4), and anti-inwardly rectifying potassium channel Kir4.1 were obtained from Millipore Corporation (Billerica, MA). Anti-β-dystroglycan antibody (clone 7D11) was obtained from Developmental Studies Hybridoma Bank (University of Iowa, Iowa City, IA). Monoclonal anti-dystrophin antibody was obtained from Millipore Corporation. A polyclonal anti-laminin-111 antibody was obtained from Sigma-Aldrich (St. Louis, MO). Antibody against cellular retinaldehyde-binding protein (CRALBP) was obtained from Abcam (Cambridge, MA).

### Western blot analyses

The retinas were homogenized on ice in Tris-buffer saline (TBS) containing 0.5% Triton X-100 and a cocktail of protease inhibitors and centrifuged at 30,000× g for 1 h at 4 °C. The supernatant was collected. Wheat germ agglutinin (WGA)-agarose was added and incubated at 4 °C overnight. Glycoproteins were eluted from the WGA-agarose and separated on a 7% sodium dodecyl sulfate–PAGE gel. The proteins were electrotransferred onto polyvinylidene fluoride membrane filters. Filters were blocked by 2% BSA in TBS, incubated with primary antibody, and then secondary antibody conjugated with horse radish peroxidase. The results were visualized with an enhanced chemiluminescence detection kit (Pierce, Rockford, IL). For the laminin overlay assay on polyvinylidene fluoride membranes, all buffers used contained 1 mM Ca^2+^ and Mg^2+^. The filter was incubated with laminin (1 μg/ml) overnight at 4 °C, washed, and detected with anti-laminin antibody as described above.

### Immunostaining

Adult mice were killed by an overdose of pentobarbital injected intraperitoneally at 400 mg/kg bodyweight, and the eyes were fixed by intracardial perfusion of 4% paraformaldehyde. After cryoprotection with 30% sucrose, the eyes were embedded in optimal cutting temperature (OCT) compound in cryomolds, cryostat sectioned in the coronal plane at 10 μm, and mounted on Superfrost plus slides (Fisher Scientific, Pittsburgh, PA). Primary and fluorescence secondary antibody incubations were performed as described previously [52,53]. Briefly, the sections were blocked with 3% BSA (BSA) in phosphate buffer (pH 7.4) for 1 h to reduced non-specific binding. Then the sections were incubated with primary antibodies diluted in 3% BSA overnight at 4 °C. Monoclonal antibodies against AQP-4, β-dystroglycan, dystrophin, CRALBP, and Kir4.1 were used at 1 to 200 dilution. Polyclonal antibody against laminin-111 was used at 1 to 1,000 dilution. After washing extensively with phosphate buffer containing 0.1% Triton X-100, the sections were then incubated with fluorescein isothiocyanate- or rhodamine-isothiocyanate-conjugated goat anti-mouse IgG or goat anti rabbit IgG at 1 to 400 dilution. After washing with phosphate buffer containing 0.1% Triton X-100, the sections were counterstained with DAPI (Sigma-Aldrich, St. Louis, MO) to show nuclei. Some slides were processed without incubation with primary antibodies as controls. Fluorescence was visualized with a Zeiss Axioskop upright fluorescence microscope.

### Electron microscopy

Newborn and adult animals were anesthetized by intraperitoneal injection of pentobarbital (400 mg/kg bodyweight). Deeply anesthetized animals were then perfused with 3.7% glutaraldehyde. The eyes, including some surrounding tissues, were then excised. The samples were postfixed in 1% osmium tetroxide and stained en bloc with 1.0% uranyl acetate, dehydrated, embedded in Poly/Bed 812 resin (Polysciences, Warrington, PA), and cut into thin sections. The sections were stained with 2.0% uranyl acetate and Reynold’s lead citrate (Polysciences). The samples were observed, and digital photographs were taken with a Tecnai T12 transmission electron microscope (FEI Company, Salem, MA).

### Preparation and atomic force microscopic analysis of the inner limiting membrane

Preparation, atomic force microscopy (AFM) force indentation experiments, and calculation of elasticity were performed as published elsewhere [54]. Briefly, retinas of P2 mice were spread onto nitrocellulose membrane filters. The retina/filter was then placed, vitreous side facing down, onto a polylysine-coated glass slide. After removal of the filter and filter-bound retinal tissue, the slides with the attached inner limiting membrane (ILM) were washed extensively with 2% Triton X-100 to remove cellular remnants. The ILM samples were immunostained for laminin to localize the transparent basement membranes (BMs) on the slides and then probed. The imaging and force indentation experiments were done by an MFP-3D AFM (Asylum Research, Santa Barbara, CA) mounted on top of an Olympus IX-71 fluorescence microscope (Olympus, Tokyo, Japan). For all experiments 100-mm long silicon-nitride cantilevers with pyramidal tips (Veeco, Inc., Santa Barbara, CA) were used that have a nominal spring constant of approximately 0.8 N/m. The elasticity was determined by the force-indentation method as previously described. Comparison of the apparent Young’s modulus between the knockouts and the wild-type controls were analyzed by ANOVA (ANOVA).

## Results

### Discontinuous inner limiting membrane in POMGnT1 knockout mice

**Figure 1:**
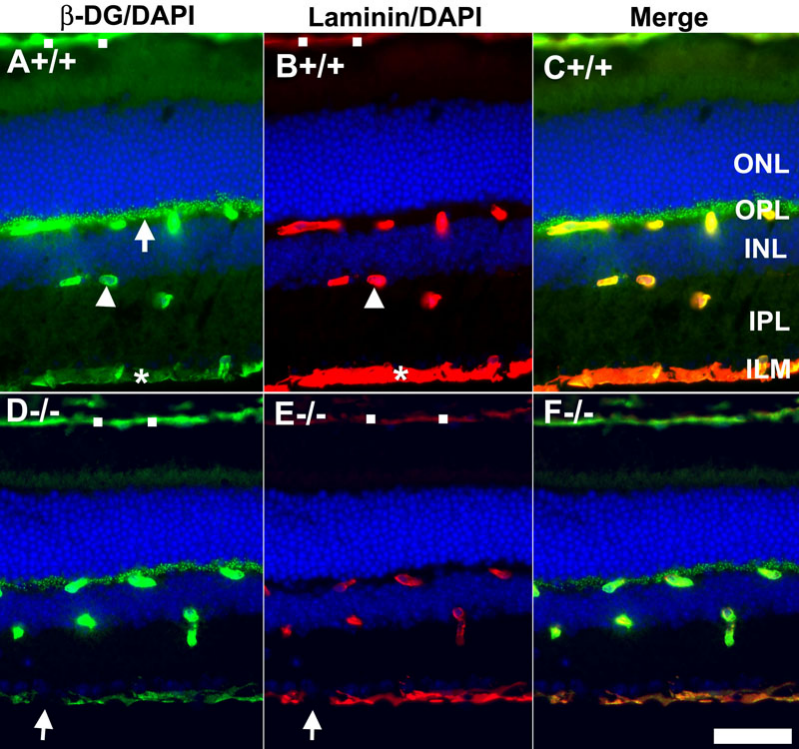
Dystroglycan distribution at the Müller glia endfeet and inner limiting membrane are disrupted in protein O-mannose N-acetylglucosaminyltransferase 1 (POMGnT1) knockout retina. Adult retinal sections were double stained with anti-β-dystroglycan (green fluorescence) and anti-laminin (red fluorescence). Wild type (**A**-**C**) and knockout (**D**-**F**) retinas were immunostained with anti β-dystroglycan (**A**, **D**) and laminin (**B**, **E**). All sections were counterstained with 4',6-diamidino-2-phenylindole to show nuclei. Merged image of **A** and **B** is shown in **C.** Merged image of **D** and **E** is shown in **F**. Note β-dystroglycan and laminin staining at the inner limiting membrane had breaks (arrows in **D** and **E**), indicating a lack of Müller glia endfeet and inner limiting membrane at these locations. Abbreviations: DG represents dystroglycan; ILM represents inner limiting membrane; INL represents inner nuclear layer; IPL represents inner plexiform layer; ONL represents outer nuclear layer; OPL represents outer plexiform layer, DAPI represents 4',6-diamidino-2-phenylindole; +/+ represents wild type; −/− represents homozygous knockout. Scale bar in **F** is equal to 50 μm.

The retina of the POMGnT1 knockout mouse was dysmorphic. We earlier reported this strain has a thinner retina and reduced density of retinal ganglion cells [25]. Western blot analysis with IIH6C4 [41,45] (an antibody that recognizes the functionally glycosylated form of α-dystroglycan) was performed. As expected, α-dystroglycan from POMGnT1 knockout mouse retina showed markedly reduced immunoreactivity, indicating that glycosylation of α-dystroglycan was affected (data not shown). To determine whether POMGnT1 deficiency, and by extension the resultant hypoglycosylation, affected dystroglycan expression in the retina, we performed immunofluorescence staining with an antibody against β-dystroglycan (Figure 1, green fluorescence). The sections were also double stained with anti-laminin to reveal the basement membrane (red fluorescence). In wild-type retinas, strong β-dystroglycan expression was detected at the inner limiting membrane (asterisk in Figure 1A), blood vessels (arrowhead in Figure 1A), the outer plexiform layer (arrow in Figure 1A), and Bruch’s membrane (open squares in Figure 1A). A weak expression of β-dystroglycan was also apparent in the inner plexiform layer. Laminin expression was detected in wild-type retinas at the inner limiting membrane (asterisk in Figure 1B), blood vessels (arrowhead in Figure 1B), and Bruch’s membrane (open squares in Figure 1B). In contrast to dystroglycan, laminin expression was not detected in the outer or inner plexiform layers (with the exception of blood vessels in these two layers). In POMGnT1 knockout retinas, the inner limiting membrane appeared discontinuous with frequent breaks (arrows in Figure 1D,E) for both β-dystroglycan and laminin immunostaining. The immunofluorescence patterns for β-dystroglycan and laminin in other retinal regions were similar to that of the wild-type retinas (compare Figure 1D–F with Figure 1A–C). We also examined the expression pattern of dystrophin, an intracellular cytoskeletal protein associated with β-dystroglycan. Its expression pattern at the outer plexiform layer and blood vessels in knockout and wild-type retinas was similar to β-dystroglycan (DG; not shown). Thus, despite reduced glycosylation, dystroglycan distribution in the knockout retina was not grossly affected, with the exception of frequent breaks in the laminin and dystroglycan labeling of the inner limiting membrane.

### Inner limiting membrane disruptions and ectopia of retinal ganglion cells in POMGnT1 knockout mice

**Figure 2:**
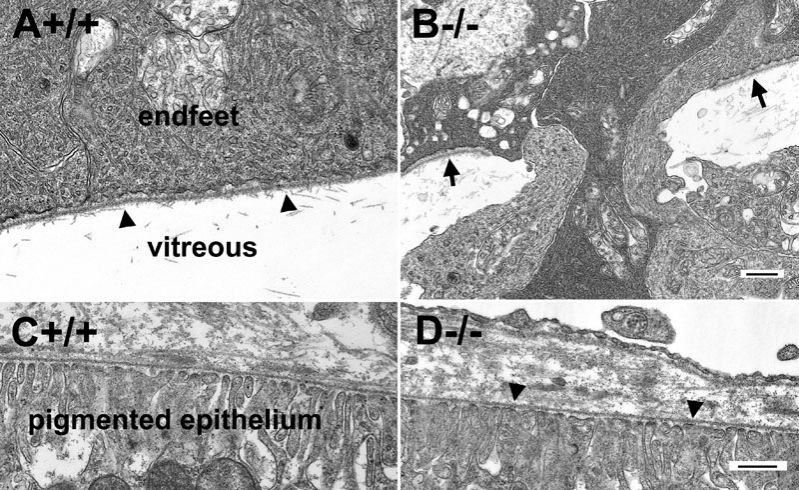
Disruptions of the inner limiting membrane in protein O-mannose N-acetylglucosaminyltransferase 1 (POMGnT1) knockout retina. Transmission electron microscopy was carried for the adult retinas. Micrographs of transmission electron microscopy of wild type (**A**, **C**) and knockout (**B**, **D**) retinas are shown. Note that the wild-type inner limiting membrane was continuous (arrowheads in **A**). Knockout inner limiting membrane was discontinuous (arrows in **B**) with protrusion of Müller glial processes into the vitreous humor. The knockout Bruch’s membrane (arrowheads in **D**) was normal. The scale bar in **B** is equal to 500 nm for **A** and **B**. The scale bar in **D** is equal to 500 nm for **C** and **D**. +/+ represents wild type; −/− represents homozygous knockout.

Loss of binding activity to extracellular matrix molecules may affect inner limiting membrane integrity because dystroglycan in the endfeet of Müller glia may serve as a receptor for basement membrane assembly. Further, frequent breaks in laminin staining suggest the possibility of inner limiting membrane disruptions. When analyzed by electron microscopy, numerous disruptions in the inner limiting membrane were confirmed for the POMGnT1 knockout retina (e.g., region between the arrows in Figure 2B). By contrast, the wild-type inner limiting membrane was continuous, displaying no breaks (arrowheads in Figure 2A). In the knockout mice, Müller glia endfeet protruded into the vitreous at sites of inner limiting membrane disruption (Figure 2B). In contrast to the inner limiting membrane, Bruch’s membrane in the knockout mice was not affected (arrowheads in Figure 2D), being similar to the wild type (Figure 2C) in displaying no apparent breaks or nearby cellular disruptions.

**Figure 3:**
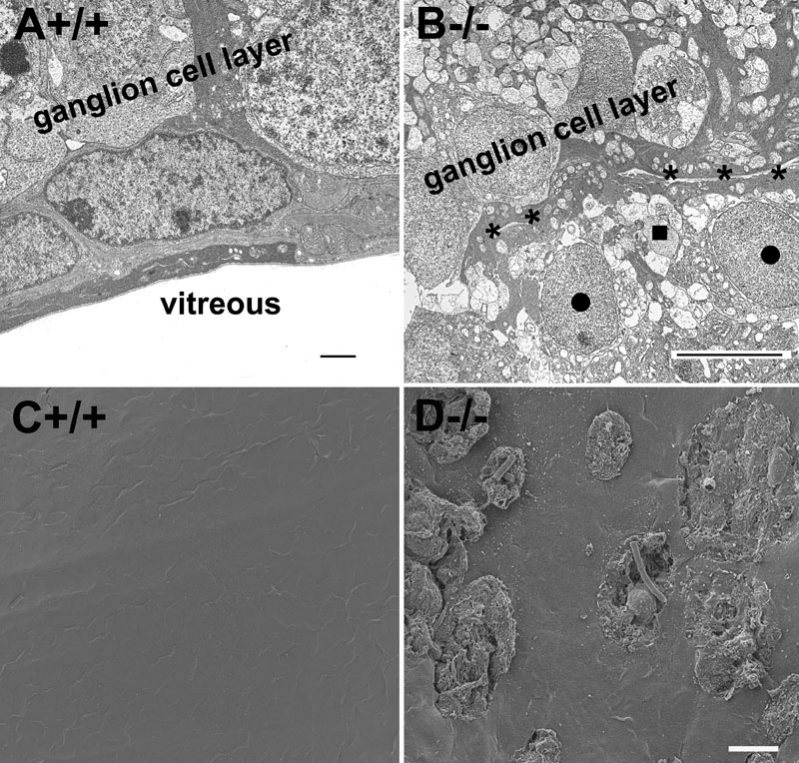
Protein O-mannose N-acetylglucosaminyltransferase 1 knockout retinas exhibit ectopia of retinal ganglion cells. Transmission and scanning electron microscopy was performed for the adult retina. Transmission electromicroscopic micrographs (**A**, **B**) and scanning electron microscopic micrographs (**C**, **D**) are shown. (**A**, **C**) show the wild type retinas. (**B**, **D**) show the knockout retinas. Note the presence of ectopic retinal ganglion cells (dark circles) and inner plexiform layer components (dark square). Asterisks indicate the location of the inner limiting membrane. The scale bar in **A** is equal to 2 μm. The scale bar in **B** is equal to 10 μm. The scale bar in **D** is equal to 10 μm for **C** and **D**. +/+ represents wild type; −/− represents homozygous knockout.

Along with protrusion of Müller glial processes into the vitreous, ectopia of some retinal ganglion cells into the vitreous was also frequently observed at the location of breached inner limiting membrane (dark circles in Figure 3B). Components of the inner plexiform layer in knockout retinas were also occasionally observed in the vitreous (dark square in Figure 3B). The ectopia of cellular processes from the retina into the vitreous was also apparent on scanning electron microscopy images of retinal whole mounts (Figure 3D). By contrast, the wild-type retina always showed a smooth inner limiting membrane surface with no ectopic cells on top (Figure 3A,C).

### Morphological and molecular changes in Müller glia

**Figure 4:**
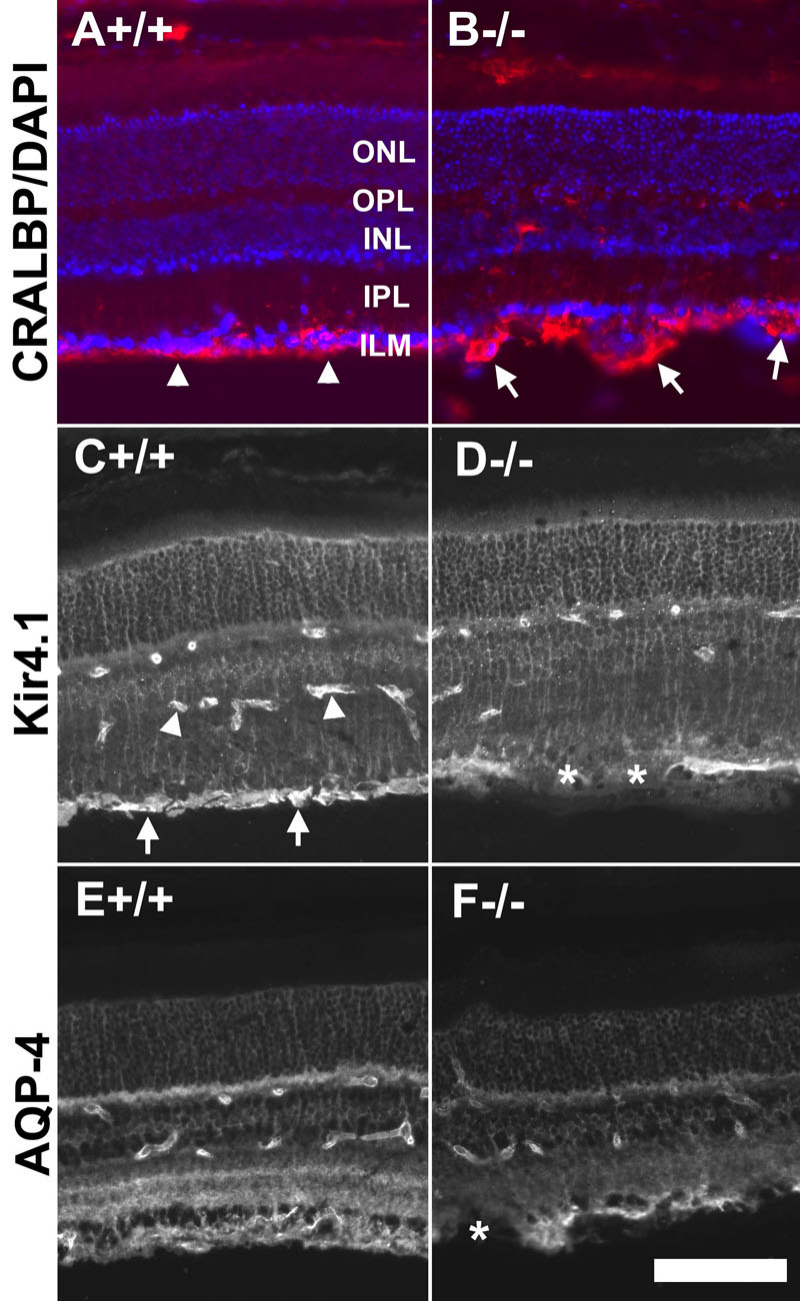
Morphological changes were observed in the Müller glia of protein O-mannose N-acetylglucosaminyltransferase 1 knockout retinas. Adult retinal sections were immunostained with antibodies against cellular retinaldehyde-binding protein (CRALBP; **A** and **B**), Kir4.1 (**C** and **D**), and aquaporin-4 (AQP-4; **E** and **F**). Fluorescence micrographs shown in **A**, **C**, and **E** are from wild type retinas and fluorescence micrographs shown in **B**, **D**, and **F** are from knockout retinas. Note the protrusion of Müller glia endfeet intro the vitreous (arrows in **B**) and reduced levels of Kir4.1 and AQP-4 at sites lacking the inner limiting membrane (asterisks in **D** and **F**). The scale bar in **F** is equal to 100 μm. Abbreviations: AQP-4 represents aquaporin-4; CRALBP represents cellular retinaldehyde-binding protein; DAPI represents 4',6-diamidino-2-phenylindole; INL represent inner nuclear layer; ILM represents inner limiting membrane; IPL represents inner plexiform layer; ONL represents outer nuclear layer; OPL represents outer plexiform layer.

To examine further the morphology of Müller glia, we screened sections with an antibody for CRALBP, a Müller glia marker (Figure 4A,B). The morphological changes in Müller glia endfeet associated with POMGnT1 deficiency described above were also evident with immunofluorescence detection of anti-CRALBP. While the Müller glia endfeet in wild-type mice formed a continuous lining at the inner limiting membrane (arrowheads in Figure 4A), the knockout Müller glia endfeet were observed to have frequent protrusions into the vitreous (arrows in Figure 4B).

**Figure 5:**
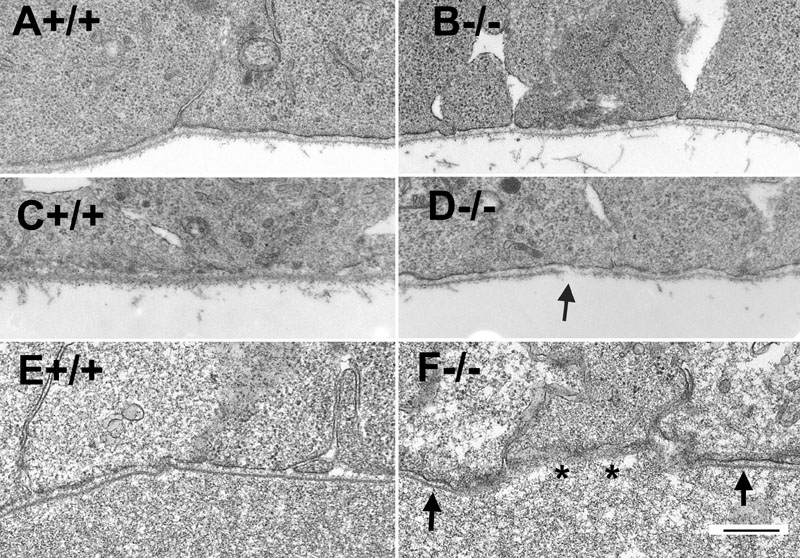
Inner limiting membrane disruptions during early eye development in protein O-mannose N-acetylglucosaminyltransferase 1 (POMGnT1) knockout mice. Electron microscopic analysis was performed on the developing retinas at embryonic day 11.5 (**A** and **B**), embryonic day 13.5 (**C** and **D**), and embryonic day 15.5 (**E** and **F**). **A**, **C**, and **E** are from wildtype retinas. **B**, **D**, and **F** are from knockout retinas. Note the broken inner limiting membrane at E13.5 (arrow in D) and the absence of the inner limiting membrane at E15.5 (asterisks in **F**) in POMGnT1 knockout mouse retina. The scale bar in **D** is equal to 500 nm. +/+ represents wild type; −/− represents homozygous knockout.

Müller glia in mouse retinas express integral membrane proteins, including the potassium channel Kir4.1 and the water channel AQP-4, at high levels. To examine whether localization of Kir4.1 and AQP-4 is affected in the knockout retina, we separately screened retinal sections with antibodies against Kir4.1 and AQP-4. In the wild-type retina, anti-Kir4.1 labeled the entire radial processes with strong labeling of the Müller glia endfeet at the inner limiting membrane (arrows in Figure 4C). In addition, blood vessels were also labeled (arrowheads in Figure 4C). In POMGnT1 knockout retina, although the Müller glia processes and blood vessels are labeled similarly to the wild type, the endfeet labeling was less robust, being characterized by particularly weak, if not altogether absent, labeling at regions with a disrupted inner limiting membrane (asterisks in Figure 4D). Altered patterns of AQP-4 distribution were also associated with POMGnT1 deficiency, with the knockout mice displaying reduced levels of AQP-4 at the inner plexiform layer (IPL) and outer plexiform layer (OPL). Like Kir4.1, there was also substantial reduction of AQP-4 at regions indicative of inner limiting membrane disruption (cf. Figure 4E and Figure 5F). Localization of the integral membrane proteins Kir4.1 and AQP-4 at Müller glia endfeet was thus disrupted in response to POMGnT1 knockout.

### Breaches in the developing inner limiting membrane precedes ectopia of Müller glia processes and retinal ganglion cells

**Figure 6:**
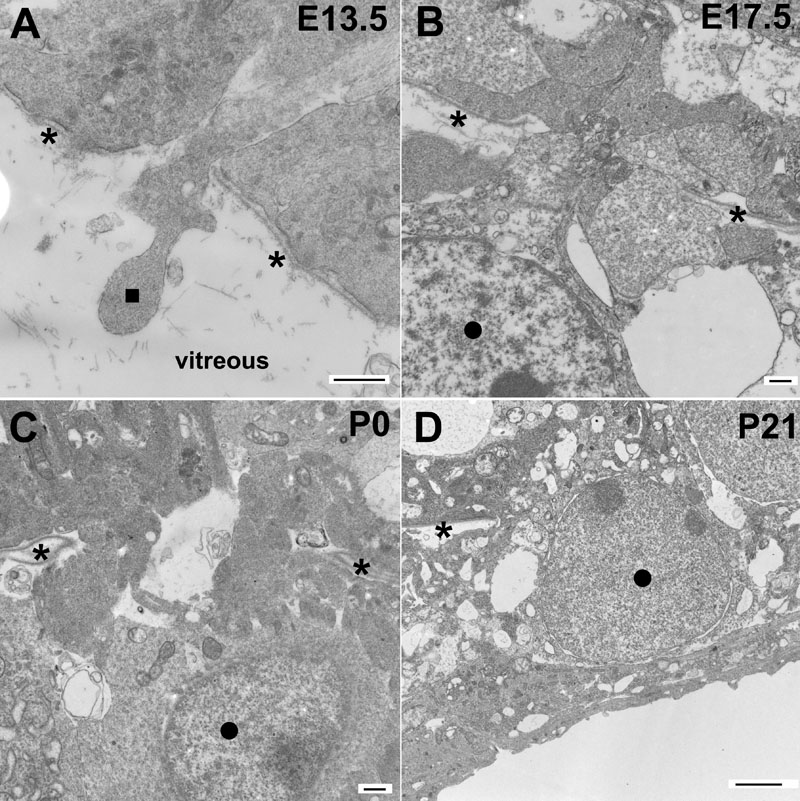
Cellular ectopia into the vitreous humor through the breached inner limiting membrane was observed at all stages after embryonic day 13.5. Protein O-mannose N-acetylglucosaminyltransferase 1 (POMGnT1) knockout retinas at E13.5 (**A**), E17.5 (**B**), P0 (**C**), and P21 (**D**) were analyzed by electron microscopy. Note the protrusion of radial glial processes (dark square in **A**) and ectopia of retinal ganglion cells (dark circles in **B**–**D**). Asterisks indicate the location of the inner limiting membrane. Abbreviations: E represents embryonic day; P represents postnatal day. The scale bars in **A**–**C** are equal to 500 nm. The scale bar in **D** is equal to 2 μm.

To explore the integrity of the inner limiting membrane during development, we performed electron microscopic analysis of the retina at several developmental stages. The inner limiting membrane at E11.5 in the knockout mouse (Figure 5B) showed the same undisrupted continuity as was seen in the wild-type mouse (Figure 5A). Breaks in the inner limiting membrane were observed as early as E13.5 (Figure 5D) and in all other older ages analyzed, including E15.5 (Figure 5F), E17.5, P0, P21, and adult. At earlier ages (E13.5 and 15.5), breaks at the inner limiting membrane were sometimes observed with (dark square in Figure 6A) or without (Figure 5D,F) associated ectopia of the glial processes into the vitreous.

At later ages (E17.5 and onward, see Figure 6B–D), all observed breaks were associated with protrusion of cellular processes from the retina into the vitreous. Ectopia of retinal ganglion cells was observed as early as E17.5 and remained evident from P0 through adult (indicated by dark circle). These results suggest that breaks in the inner limiting membrane associated with POMGnT1 deficiency temporally precedes, and thus could be the causative factor of, atypical protrusion of cell processes and retinal ganglion cells into the vitreous.

### Reduced stiffness of the inner limiting membrane

**Figure 7:**
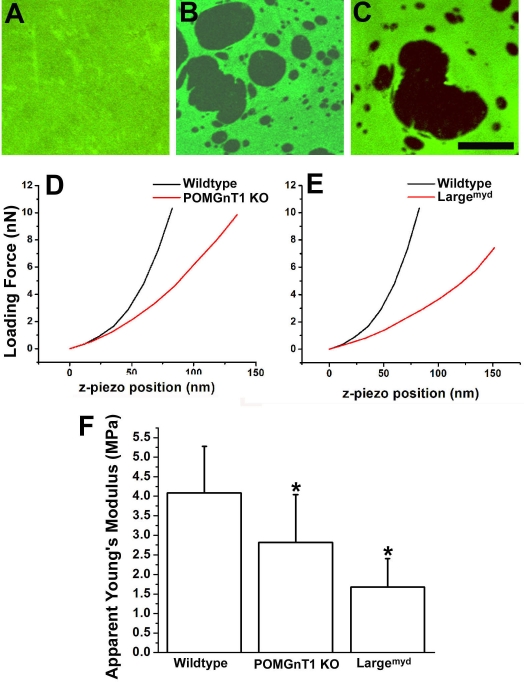
Inner limiting membranes of protein O-mannose N-acetylglucosaminyltransferase 1 (POMGnT1) knockout and Large^myd^ mice exhibit reduced elasticity. Flatmounted inner limiting membranes from postnatal day 2 (P2) newborn mice were immunostained with anti-laminin. AFM indentation experiments were performed to determine the Young’s modulus. Micrographs of laminin immunofluorescence staining of wild type (**A**), protein O-mannose N-acetylglucosaminyltransferase 1 knockout (**B**) and Large^myd^ mice (**C**) are shown. Note that the mutant inner limiting membranes showed many holes (**B** and **C**). **D** and **E** are representative force displacement curves for indentation experiments on the inner limiting membranes of protein O-mannose N-acetylglucosaminyltransferase 1 knockout and Large^myd^ mice. Apparent Young’s modulus measurements from inner limiting membranes isolated from 4 mice for each genotype are shown in **F**. The bars represent means with standard deviations (p<0.01, ANOVA). When compared to the controls, significantly reduced Young’s modulus was observed for both POMGnT1 knockout (p<0.01) and Large^myd^ (p<0.01) inner limiting membranes (post hoc Student’s *t*-test). Asterisks in **F** indicate p<0.01. These results indicated that the inner limiting membranes of protein O-mannose N-acetylglucosaminyltransferase 1 knockout and Large^myd^ mice exhibited reduced elasticity compared to the wild type. MPa represents megapascal. nN represents nanonewton. The scale bar in **C** is equal to 25 μm.

To determine whether the biomechanical properties of the knockout inner limiting membrane is changed, we isolated the inner limiting membrane from POMGnT1 knockout and Large^myd^ mice since Large^myd^ mice also exhibit disruptions of the inner limiting membrane [27]. When stained with anti-laminin, the flatmounted inner limiting membrane from the wild type showed a continuous undisrupted immunofluorescence (Figure 7A). By contrast, the inner limiting membranes of POMGnT1 knockout and Large^myd^ mice exhibited many holes (Figure 7B,C). These holes are caused by the disruptions in the inner limiting membrane, as described above (Figure 2, Figure 3, and Figure 6). To measure the Young’s modulus, force indentation experiments were performed on four inner limiting membrane preparations from each genotype, using AFM (Table 1). Figure 7D,E show representative curves of AFM loading force versus the z-piezo position for POMGnT1 knockout and Large^myd^ inner limiting membranes. These measurements showed that the apparent Young’s modulus (Figure 7F) for the wild-type inner limiting membrane was 4.08±1.19 MPa (mean±standard deviation; n=4). The Young’s moduli of the inner limiting membranes from POMGnT1 knockout and Large^myd^ mice were significantly reduced (2.82±1.22 and 1.68±0.73 MPa, respectively; p<0.01, ANOVA; n=4 each). AFM thickness measurement did not reveal significant differences between the knockouts and the wild type (data not shown). These results indicated that POMGnT1 knockout and Large^myd^ inner limiting membranes exhibited reduced stiffness.

**Table 1:** Measurement of apparent Young’s modulus (MPa) of inner limiting membranes from wildtype, POMGnT1 knockout and Large^myd^ mouse retinas.

**Sample #**	**Wildtype**	**POMGnT1 knockout**	**Large^myd^**
1	4.11±0.90	2.83±0.95	1.66±0.56
2	4.01±1.02	2.75±1.02	1.90±0.93
3	3.76±1.14	3.10±0.84	1.69±0.49
4	4.46±1.41	2.59±0.79	1.46±0.54

Elasticity was measured at 100 random locations from each of the four inner limiting membrane (ILM) samples of every genotype. Means±standard deviations were then calculated.

## Discussion

The POMGnT1 knockout mice were previously reported to exhibit a thinner retina with reduced density of retinal ganglion cells [25]. In this paper, we report that some ganglion cells were located ectopically on the vitreous side of the inner limiting membrane. The retinal ganglion cell ectopia is a developmental event caused by physical breaches in the inner limiting membrane that occur starting at E13.5. Interestingly, the basement membrane of the pigmented epithelium, the Bruch’s membrane, is not affected by POMGnT1 deficiency. Thus, with respect to basement membrane biology in the retina, POMGnT1-mediated glycosylation events are apparently selectively required for maintaining the structural integrity of the inner limiting membrane.

Ectopia of nerve cells through the basement membrane into the mesenchymal tissue also occurs in the cerebral cortex and the cerebellum of POMGnT1 knockout mice [53,55]. Does the breached basement membrane permit the movement of cells to ectopic locations? Or do the migrating cells/processes penetrate the intact basement membrane, causing the breaches in the basement membrane? At all developmental stages analyzed for the cerebral cortex and the cerebellum, disrupted basement membrane was always associated with protrusion of radial and Bergman glia, respectively, and associated with ectopia of cerebral cortical neurons and cerebellar granule cells [53]. It was not possible to discern the causal relationship of the breaches in the pial basement membrane and the protrusion of cell processes and migrating neurons. The current investigation of retinas in POMGnT1-deficient mice, however, indicated that at their earliest manifestations, breaches in the inner limiting membrane were not always associated with protrusion of cells or cellular processes. Such associations were invariably observed at later developmental points. These findings are consistent with the model that neural ectopias follow breached basement membranes.

A broken inner limiting membrane is also observed in some other animal models. In the natural mutant of *Large*, Large^myd^ mice, a gene involved in glycosylation of α-dystroglycan [27], breached inner limiting membrane and ectopia of retinal ganglion cells were observed. Mutations in the laminin β2 chain, a component of the basement membrane, cause congenital nephritic syndrome with abnormal retina [56]. Mouse knockout of β2 laminin also exhibits broken inner limiting membrane along with altered morphology of photoreceptor outer segments, synaptic disruption in the outer plexiform layer (OPL), and disoriented Müller glia [57-59]. Earlier studies showed that survival of retinal ganglion cells is dependent on the integrity of the inner limiting membrane [60]. Thus, the reduced retinal ganglion cell density found in POMGnT1 knockout mice may be directly related to the disruptions in the inner limiting membrane.

Although it is presumed that abolished binding between the hypoglycosylated α-dystroglycan and the laminin of the basement membrane is involved in the observed retinal abnormalities, the mechanisms that cause basement membrane breaches in various congenital muscular dystrophy models are not clear. Through analysis with AFM, we found that the inner limiting membranes of POMGnT1 knockout and Large^myd^ mice have significantly reduced stiffness. Thus, reduced physical strength of the inner limiting membrane is at least a contributing factor in their disruptions. Major components of the inner limiting membrane are produced by the lens and ciliary body, secreted into the vitreous, and assembled at the retinal inner surface [61-63]. Because extracellular matrix receptors, particularly integrin and dystroglycan, at the cell surface are instrumental in assembly of the basement membrane [64], reduced binding to laminin might result in the assembly of a defective basement membrane that is mechanically weaker.

The key molecule mediating the functions of congenital muscular dystrophy genes is believed to be α-dystroglycan. Dystroglycans (α and β) are required for normal retinal development, as morpholino-mediated knockdown of dystroglycan expression in the developing *Xenopus* eye results in severe lamination defects [65]. In the mammalian retina, dystroglycan is highly expressed by photoreceptors at the presynaptic terminal in the outer plexiform layer and by Müller glia cell endfeet at the inner limiting membrane [66-73]. Knockout of dystroglycan in the developing mouse embryo results in a thinner retina with a disrupted inner limiting membrane [74]. High expression of dystroglycan in the presynaptic terminals of photoreceptors and inner limiting membrane suggests that glycosylation defects of α-dystroglycan may affect these two sites of the retina. Indeed, as demonstrated in the current study, a primary site of defect in POMGnT1 knockout retina is the inner limiting membrane where breaches resulted in ectopia of retinal ganglion cells and Müller glia processes.

**Figure 8:**
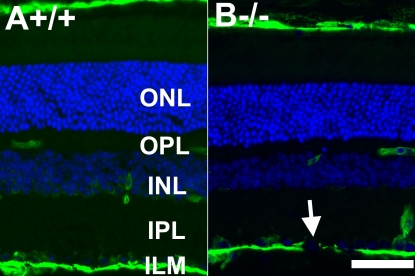
Breached inner limiting membrane in Large^myd^ mouse retina. Adult wild type (**A**) and Large^myd^ (**B**) mouse retinal sections were double stained with anti-laminin (green fluorescence) and counterstained with 4',6-diamidino-2-phenylindole to show nuclei (blue fluorescence). +/+ represents wild type; −/− represents homozygous mutant. Note the discontinuity of the inner limiting membrane in Large^myd^ (−/−) mouse retina. The scale bar in **B** is equal to 50 μm. Abbreviations: INL represent inner nuclear layer; ILM represents inner limiting membrane; IPL represents inner plexiform layer; ONL represents outer nuclear layer; OPL represents outer plexiform layer.

It is of some interest to compare the phenotypes between POMGnT1 knockout and Large^myd^ mice. Both inner limiting membranes are breached with reduced stiffness, resulting in ectopia of retinal ganglion cells. While the majority of the laminin staining at the inner limiting membrane of Large^myd^ retina is lost, according to a previous report [75], abnormal laminin staining in POMGnT1 knockout mice was only observed at sites where the basement membrane was apparently disrupted. This could be due to technical differences in the antibodies used for staining. We therefore stained Large^myd^ mouse retinal sections with anti-laminin antibody. Similar to POMGnT1 knockout mice, significant laminin immunostaining at the inner limiting membrane was observed in Large^myd^ mouse retina. However, laminin immunostaining was notably absent in areas where the inner limiting membrane was apparently disrupted (see arrow in Figure 8B). In areas free of breach, the laminin staining of the inner limiting membrane was continuous in both mouse models.

Laminin interaction with the dystroglycan complex is known to regulate the distribution of Kir4.1 and AQP-4 [76,77]. In normal retina, both of these membrane integral proteins are present throughout the radial extent of Müller glial but are present at particularly high levels at the endfeet. Astrocytic endfeet at the blood vessels also highly express both of these channel proteins. As expected, their localization in POMGnT1 knockout retina is dependent on the integrity of the inner limiting membrane. In regions where the inner limiting membrane is normal, the presence of both channels remains comparable to the controls. However, at sites of inner limiting membrane breaches, their levels are substantially reduced. These results suggest that the mechanism of POMGnT1-dependent disruptions in retinal organization associated with the inner limiting membrane involves hypoglycosylation of the dystroglycan/laminin complex.

Interestingly, immunofluorescence labeling revealed that dystroglycan distribution in the outer plexiform layer of POMGnT1 knockout mice remains largely normal. Nevertheless, the defective electroretinogram b-wave of POMGnT1 knockout mice suggests that O-mannosyl glycosylation may play a role in synaptic transmission in the outer retina [25]. Interpretation of this result, however, is complicated by anatomic defects observed in the null knockout mice. Conditional knockout mice, where O-mannosyl glycosylation can be knocked out with (presumably) a minimum of developmental malformations, will enable an improved understanding of how POMGnT1 and O-mannosyl glycosylation contribute to retinal development, structure, and function.
